# The relationship between fatigue, sleep quality, resilience, and the risk of postpartum depression: an emphasis on maternal mental health

**DOI:** 10.1186/s40359-023-01043-3

**Published:** 2023-01-13

**Authors:** Baian A. Baattaiah, Mutasim D. Alharbi, Nouf M. Babteen, Haneen M. Al-Maqbool, Faten A. Babgi, Ashar A. Albatati

**Affiliations:** Department of Physical Therapy, Faculty of Medical Rehabilitation Sciences, King Abdulaziz University, P.O. Box 80200, Jeddah, 21589 Saudi Arabia

**Keywords:** Postpartum, Depression, Fatigue, Sleep quality, Resilience, Women’s health

## Abstract

**Background:**

Several factors can contribute to the development of postpartum depression (PPD) and negatively affect mothers’ mental and physical well-being. The objective of this study was to determine the relationship between fatigue, sleep quality, resilience, and the risk of PPD development.

**Methods:**

A cross-sectional study was conducted using an online questionnaire distributed to mothers during their postpartum period. The risk of PPD was assessed using the Edinburgh Postnatal Depression Scale (EPDS), postpartum fatigue (PPF) was assessed using the Fatigue Severity Scale (FSS), sleep quality was assessed using the Pittsburgh Sleep Quality Index (PSQI), and resilience was assessed using the Brief Resilience Scale (BRS). The Pearson correlation coefficient was calculated to determine the relationship between the study variables. Simple and multiple linear regression analyses were performed to explain the contributions of PPF, sleep quality, and resilience as independent predictors of PPD development.

**Results:**

A total of 1409 postpartum women were included in the analysis, with 75% of the participants reporting a risk of PPD, 61% reporting PPF, 97% reporting having sleep problems, and 36% being in the “low resilience level” category. In terms of correlations, the scores of FSS and the PSQI showed moderate positive relationships with the EPDS scores (r = 0.344 and r = 0.447, respectively, *p* = .000). The BRS scores were negatively associated with the EPDS scores (r = −0.530, *p* = 0.000). Fatigue, sleep quality, and resilience were predictors of depressive symptoms (β = 0.127, β = 0.262, and β = −0.393, respectively, R^2^ = 0.37, *p* = 0.000). The association remained significant in the regression model after adjusting for mother’s age, mother’s BMI, child’s age, smoking status, full-term pregnancy, having a chronic disease, and taking anti-depressant.

**Conclusions:**

Mothers with higher levels of fatigue, poor sleep quality, and low resilience levels were at high risk of developing PPD. Healthcare providers should identify these factors and thus set better rehabilitation goals to improve overall maternal health.

## Background

One of the most common complications among postpartum women is postpartum depression (PPD). According to established diagnostic criteria, PPD is defined as an episode of non-psychotic depression that occurs within one year after giving birth [1]. According to the World Health Organization (WHO), depression is “a disease characterized by persistent melancholy and a lack of interest in activities that one ordinarily enjoys, as well as an incapacity to carry out everyday tasks, for at least two weeks” [2]. There are three main types of depression that may occur during the postpartum period: the postpartum blues, PPD, and postpartum psychosis. Postpartum blues, or the “baby blues,” refers to the common range of physical discomforts and symptoms experienced by mothers in the first week or so following childbirth. Estimates place the prevalence of the postpartum blues at between 26 and 84% of all new mothers [3, 4]. Dysphoric mood, sobbing, mood lability, anxiety, sleeplessness, loss of appetite, and irritability are all signs of the postpartum blues. Postpartum depression is generally independent of the blues, but having the postpartum blues does indicate a risk of PPD [5]. Postpartum depression, which affects around one out of every ten new mothers, is significantly more severe disorder than the postpartum blues. Women who have previously had PPD have a 30% higher risk of having PPD after subsequent deliveries. Women with PPD may experience alternating highs and lows, frequent sobbing, irritability, and fatigue, as well as feelings of guilt and fear and an incapacity to care for their infant or themselves. Symptoms can range from moderate to severe and can emerge immediately after the mother gives birth or over time, and last up to a year later [6]. Postpartum psychosis is the most severe type of depression that occurs during postpartum. The prevalence of postpartum psychosis is one or two per 1000 births, and women with postpartum psychosis have different symptom patterns than women with psychosis unrelated to childbirth. Postpartum psychosis is characterized by mood swings, disorientation, and significant cognitive impairment, as well as odd behavior, sleeplessness, and hallucinations. One particularly dangerous example of the symptoms of postpartum psychosis is the delusion of altruistic homicide, in which mothers believe they are saving their children from a fate worse than death by killing them [5].

Because PPD is common among new mothers [7], research should focus on improving health and quality of life for postpartum women by investigating the factors that could contribute to the development of PPD. Postpartum depression can be attributed to several physical, physiological, and psychosocial predisposing factors. However, little is known about the contributions of fatigue, sleep disturbance, and resilience to the development of PPD. Fatigue is a universal problem that is not always related to medical diagnoses or therapeutic treatment [8]. It is characterized as an imbalance between activity and rest that cannot be relieved by a single period of sleep [9]. Fatigue is considered to be one of the most common conditions impacting women during the postpartum period. It affects 63.8% of new mothers and may be due to several conditions that may have occurred during this critical period, such as anemia, infection, inflammation, and thyroid dysfunction [10, 11]. Other studies have suggested that maternal hormonal changes also contribute to fatigue in the postpartum period [12]. Evidence suggests that persistent fatigue can increase a woman’s risk of PPD [13]. A study by Corwin et al. assessed postpartum fatigue (PPF) in relation to depressive symptoms and stress. The results revealed significant correlations between PPF and symptoms of PPD in the first three weeks, with fatigue at two weeks postpartum being the most predictive variable for the development of PPD [14]. However, extensive evidence should be gathered to confirm PPF as an independent predictor of the PPD symptoms experienced by mothers.

Sleep quality is another concern among postpartum women. Sleep disturbance can occur in the form of deprivation, which refers to a lack of sleep, or fragmentation, which refers to numerous awakenings after an individual falls asleep [15]. Sleep is both a basic physiological necessity and a complicated physiological process that restores physical agility and energy. The primary cause of postpartum sleep disturbance is infant signaling during the night [16]. Sleep disturbance in postpartum women presents significant challenges, including impairing the performance of everyday tasks for several months after childbirth [17]. In 2019, Christian et al. [18] assessed sleep quality in 133 nulliparous and multiparous African American and white women during each trimester of pregnancy and at four to eleven weeks postpartum. The study found that all participants suffered from poor sleep quality throughout their pregnancies and during the early postpartum period. In 2018, Okun et al. [19] assessed women’s sleep quality at six months postpartum to identify associations between sleep quality and symptoms of depression and anxiety throughout pregnancy and postpartum. Their results suggest that poor sleep quality is linked with increased depression and anxiety symptoms in women at six months postpartum. Moreover, Lewis et al. used both the Pittsburgh Sleep Quality Index (PSQI) and the Patient Health Questionnaire-9 (PHQ-9) to investigate the relationship between changes in self-reported sleep patterns and depressive symptoms from six weeks to seven months postpartum among American women who were at a high risk of developing PPD. The study revealed that those who are at a high risk of PPD are also at a higher risk of developing depressive symptoms later in their postpartum period if sleep issues persist or improve very minimally over time [20]. While these studies produced insightful results regarding the relationship between sleep disturbance and the risk of developing PPD, there was heterogeneity in terms of the sample characteristics and the timepoints at which PPD symptoms were studied.

Resilience is a psychological factor that refers to “the personal qualities that enable one to thrive in the face of adversity” [21]. Masten and Cicchetti [22] emphasized the importance of resilience, which they defined as the potential ability of an individual to successfully adjust to situations that threaten functions, survival, or growth. They also delineate the promotive and protective impact of resilience for human development. Conceptualizations of resilience generally include the utilization of specific coping strategies, such as social support and religious participation, and, in a broader sense, having the ability to overcome difficulties [23]. Notably, resilience is a factor in the psychological outcomes of women with histories of maltreatment, including their risk of depression [24, 25]. For instance, Sexton et al. [26] in 2015, found that resilience was connected to decreased psychopathology and increased well-being in mothers. Unfortunately, limited research has been conducted on the independent role of resilience in the risk of PPD.

There is a paucity of studies investigating the relationships between fatigue, sleep quality, and resilience and the risk of developing PPD among women who live in Saudi Arabia. Despite variations in theoretical models, it has been shown that depression, fatigue, sleep quality, and resilience contribute to activity participation, as well as that a lack of participation may eventually compromise women’s quality of life and overall health [27–32]. Therefore, this study aimed to investigate the role of fatigue, sleep quality, and resilience in the risk of developing PPD. The results of our study introduce a new concept based on improving the quality of life for women during postpartum, as well as women’s health overall. They also add to the fundamental knowledge pertaining to the healthcare services that should be provided to women during this critical time. The findings emphasize developing early rehabilitation, interventional, and management strategies in order to minimize the risk of PPD development and thus improve maternal health.

## Methods

### Study design and population

This was a cross-sectional study design that involved an electronic survey. The questionnaires attached to the survey were generated and distributed via various social media platforms (Email, Twitter, and WhatsApp) targeting mothers who live in Saudi Arabia. The estimation of the sample size required for this study was calculated using OpenEpi.com [33]. According to the General Authority of Statistics in Saudi Arabia for the total adult female population in 2021, with a confidence level set at 95%, a margin of error at 5%, and an anticipated frequency at 50%, the estimated sample size was 385 participants.

The study was carried out between the months of February and May of 2022. Women who were aged 18–45 years old [34–38] and were in the postpartum period, defined as from two weeks up to one year after giving birth, were included in the study. Mothers who had a history of psychological illness before giving birth that might have interfered with our study findings were excluded. The survey was completed by a convenience sample consisting of 1443 participants. Thirty-four participants were excluded from the study, and a total of 1409 participants who met the study’s inclusion criteria were included in the final analysis.

This study was conducted in accordance with the guidelines proposed in the Declaration of Helsinki and reviewed and approved by the Ethics Committee of the Faculty of Medical Rehabilitation Sciences (FMRS), King Abdulaziz University, Jeddah, Saudi Arabia (FMRS-EC2022-006). At the beginning of the survey, the study’s protocol and procedure and the participants’ rights were all explained. The informed consent was also obtained from all participants before they begin filling out the survey. The email address of the principal investigator was provided at the beginning of the survey in case participants had any questions related to the study.

### Study instrument/measures

The study was conducted using mostly quantitative methods. The link to the survey was distributed via email and the social media networking sites, WhatsApp, and Twitter. Women who were less likely to access these social networks were recruited and encouraged to complete the online survey via direct phone/text contact. All participants were asked to complete the online survey, which consisted of several socio-demographic and health-related questions, along with the Edinburgh Postnatal Depression Scale (EPDS), the Fatigue Severity Scale (FSS), the Pittsburgh Sleep Quality Index (PSQI), and the Brief Resilience Scale (BRS). All participants were encouraged to share the survey link with their families and friends. A reminder to participate in the survey was sent biweekly to maximize the response rate during the data collection period. The time required to fill out the online questionnaires did not exceed five to seven minutes from start to finish.

### Socio-demographic and health- and childbirth-related questions

All participating mothers were asked to indicate their age, weight, height, educational level, nationality, and geographic location. Data related to academic achievements, marital status, employment status, and number of hours worked per week were also collected. Participants were asked to indicate their comorbidities and any history of psychological illness, such as depression, before giving birth. They were also asked about the following: whether they were currently pregnant, the age of their youngest child, the number of children they have, whether their youngest child has a medical condition, the mode of delivery their youngest child had, whether there were any complications with this delivery, and whether they had a personal caregiver during their postpartum period. Finally, the participants were asked if they regularly exercised and if they smoked.

### Edinburgh postnatal depression scale (EPDS) [39]

The EPDS, a ten-item scale developed by Cox et al., was originally designed to identify PPD disorders, which are distressing disorders that last longer than the “blues” but are less severe than postpartum psychosis. Each item on the EPDS is scored on a 4-point scale between 0 and 3 based on the severity of the symptoms, and total scores can range from 0 to 30. The EPDS is the most widely used PPD screening tool in both the pre- and postnatal periods, though it has been proven to be more robust when used in the postnatal period [40]. The EPDS has been translated into and validated in 18 languages, including Arabic [41]. The Arabic version of the EPDS has a Cronbach’s alpha coefficient of 0.84, indicating good internal consistency. The questionnaire is generally intended to screen participants’ depression levels in order to perform additional assessments and treatment recommendations. A cutoff value of ≥ 12 has been shown to indicate good specificity for postpartum depressive symptoms among Arabic-speaking population [42].

### Fatigue severity scale (FSS) [43]

The FSS was established by Krupp et al., who defined fatigue as “a sense of physical tiredness and lack of energy, distinct from sadness or weakness” [44]. It is the most commonly used means of measuring fatigue in people with chronic conditions. The original FSS is a unidimensional nine-item questionnaire consisting of statements rated on a seven-point Likert scale ranging from 1 (strongly disagree) to 7 (strongly agree) [43]. The FSS score is calculated using the mean of the item scores. The fatigue cut-off score was established as ≥ 4, while the non-fatigue score was (FSS < 4.0) [45–48]. The FSS corresponds highly with other tiredness measures, and it has been shown to distinguish between healthy and chronically ill individuals [43, 49–53]. The FSS was translated into Arabic language and the questionnaire is valid for use by Arabic-speaking populations [48]. The reliability of the Arabic version of FSS shows good internal consistency when measured via Cronbach’s alpha (α = 0.84).

### Pittsburgh sleep quality index (PSQI) [54]

The PSQI was developed by Buysse and consists of 19 items divided into seven components that assess the seven characteristics of sleep during the last month: sleep quality, sleep onset latency, sleep duration, sleep efficiency, sleep disturbances, sleeping medication use, and daytime dysfunction. Each component is scored between 0 (no difficulty) and 3 (severe difficulty), with a total score between 0 and 21 points. Generally, the higher scores indicate worse sleep quality. Poor sleep quality is particularly indicated by a total score of 5 or higher [55, 56]. The Arabic version of the PSQI demonstrated adequate validity and acceptable reliability (α = 0.65) for assessing sleep quality in Arabic-speaking people [57].

### Brief resilience scale (BRS) [58]

The BRS was originally developed by Smith et al. The scale is valid and reliable for assessing human resilience. It consists of six items with potential responses of: 1 = strongly disagree; 2 = disagree; 3 = neutral; 4 = agree; and 5 = strongly agree. Total scores range from 6 to 30 points. The scale is scored by first reverse the coding of the items 2, 4, and 6 and then taking the mean of all items scores. For adults and in general, poor resilience is indicated by a score ≤ 2.95, and high resilience is indicated by a score ≥ 3.99. For females, poor resilience is indicated by a score ≤ 2.87, and high resilience is indicated by a score ≥ 3.91 [58, 59]. The translation of the BRS into Arabic language was performed according to international standards (forward and backward translations). The content validity was then assessed by ten experts in the field, and the content validity index (CVI) was measured to be 0.8. The face validity was assessed with 32 participants taken from the intended population, and the face validity index (FVI) was calculated as 0.87. A reliability testing was conducted to examine the internal consistency of the Arabic translated version of BRS. The results yielded an excellent Cronbach’s alpha coefficient value (α = 0.98).

### Data analysis

All collected information were screened for missing data, outliers, and normality. In the data-cleaning process, we did not identify any missing data related to the study’s main variables. The Shapiro–Wilk test was performed to test the normality assumption regarding the data. Data are presented as means and standard deviations (SDs) for continuous variables and as frequencies and percentages for categorical variables. The Pearson’s correlation coefficient was utilized to examine the relationship between the scores of the EPDS and the FSS, the PSQI, and the BRS. Simple and multiple linear regressions were used to investigate the predictive factors for EPDS scores. Statistical significance was set at *p* ≤ 0.05. The collected data were analyzed using the Statistical Package for the Social Sciences (SPSS) Version 26 (IBM Corp., Chicago, IL, USA).

## Results

### Participants’ socio-demographic and health-related characteristics

Of the 1443 participants who responded to the survey, 1409 (98%) were included in the analysis. The socio-demographic characteristics of all participants are presented in Table 1. The mean age of the participants was 29 ± 5, while the mean body mass index (BMI) was 26 ± 5. The majority (59%) of the participants were between the ages of 20 and 29 years old, and 45% were considered within the normal BMI range. The findings revealed that most of the participants (roughly 1348 (96%)) were married. More than half (66%) of the participants held a bachelor’s degree, and 65% reported they were unemployed. In regard to nationality, 1196 (85%) of the participants were Saudi citizens, and 33% resided in the Macca region.

**Table 1:** Participants’ Socio-demographic Characteristics (N = 1409)

Variable	Categories	Frequency (n)	Percentage (%)
Age	< 20	28	2.0
	20–29	834	59.2
	30–39	508	36.1
	40–45	39	2.8
BMI	Underweight	63	4.5
	Normal	639	45.4
	Overweight	464	32.9
	Obese	243	17.2
Marital status	Married	1348	95.7
	Divorced	27	1.9
	Married with complicated issues or separated without being divorced	34	2.4
Educational level	No certificate	8	0.6
	Secondary or less	231	16.4
	Diploma	106	7.5
	Bachelor	933	66.2
	Master	117	8.3
	Ph.D	14	1.0
Current work	Government employee	146	10.4
	Unemployed	912	64.7
	Free business	71	5.0
	Student	177	12.6
	Private sector employee	103	7.3
Nationality	Saudi	1196	84.9
	Non-Saudi	213	15.1
Region of residence	Mecca	466	33.1
	Riyadh	300	21.3
	Eastern province	191	13.6
	Medina	89	6.3
	Asir	85	6.0
	Other	278	19.7

Data are presented as frequency (n) and percentage (%)

*BMI* Body Mass Index

Data related to participants’ health and childbirth conditions are presented in Table 2. The majority (95%) of our participants were non-smokers, and about 1265 (90%) reported no chronic diseases. Data revealed that 69% of participants had undergone a normal vaginal delivery, with roughly 54% reporting no difficulties during delivery. A total of 1269 (90%) reported full-term pregnancy, with the mean age of the children being 8 ± 4 and 64% having a child older than six months of age, while only 8% reported child health problems. The majority of the participants (63%) reported having received family assistance during their postpartum period. Only 3% of the participants reported taking antidepressants after childbirth. The data further revealed that the majority of mothers (91%) were not pregnant while responding to the survey and 671 (49%) of the participants had only one child. Most mothers (61%) reported that they exercised regularly, with 44% indicating that they exercise at home and 51% reporting that they perform light-intensity exercise.

**Table 2:** Participants’ Health and Childbirth Characteristics (N = 1409)

Variable	Categories	Frequency (n)	Percentage (%)
Smoking status	Yes	68	4.8
	No	1,341	95.2
Chronic diseases	Yes*	144*	10.2
	No	1265	89.8
Delivery mode	Normal	972	69.0
	Caesarean section	437	31.0
Delivery difficulty	Yes	196	13.9
	To some extent	454	32.2
	No	759	53.9
Full-term pregnancy**	Yes	1269	90.1
	No	139	9.9
Child age (months) *(mean* = *8, SD* = *4)* **	6 or less	511	36.3
	> 6	897	63.7
Total number of children**	1	671	49.4
	2	361	26.6
	3	186	13.7
	4	88	6.5
	5	44	3.2
	6	7	0.5
Child health problems	Yes	109	7.7
	No	1,300	92.3
Having assistance/home helper	Family assistance	885	62.8
	Household aid (hourly)	41	2.9
	Nursery	10	0.7
	Helper at home	126	8.9
	No help	347	24.6
Taking antidepressants	Yes	36	2.6
	No	1,373	97.4
Currently pregnant	Yes	129	9.2
	No	1,280	90.8
Regular exercise	Yes	860	61.0
	No	549	39.0
Place of exercise**	Gym club	115	13.5
	At home	378	44.4
	Personal trainer at home	11	1.3
	Regular/routine activities	348	40.8
Intensity of exercise**	Light	432	50.8
	Medium	403	47.4
	High	16	1.9

Data are presented as means, standard deviations (SDs), frequencies (n), and percentages (%)

*Of these, 10% suffered from gastrointestinal diseases, 24% allergies, 21% lung/chest diseases, 12% diabetes mellitus, 7% hypertension, 3% nervous system diseases, 1% cardiovascular diseases, 1% musculoskeletal diseases, and 20% other diseases

**Total frequencies may not add up to 1409, due to missing data (questions either left unanswered or responded to unclearly)

### Data related to the study measures

Data related to the study measures are presented in Table 3. The mean score on the EPDS was 16.12, with a total of 1051 (75%) of the participants reporting risk of PPD. In regard to the FSS, the mean score was 4.30, with fatigue reported by around 61% of the participants. The mean PSQI score was 10.37, and 97.2% of participants reported that they had experienced sleep issues; the majority of these had a response in the range of 1–3, and component 4 (sleep efficiency) had the highest mean response (3) among the components, indicating general sleep quality issues. Component 7 (daytime dysfunction) had the lowest mean response (0), indicating a lack of general daytime dysfunction. The data further revealed that the mean score on the BRS was 3.18, with almost 501 (36%) of all participants were within a low resilience level category. The results also showed that 365 (26%) of mothers reported depressive symptoms, along with high levels of fatigue and poor sleep and a low level of resilience.

**Table 3:** Descriptive Statistics of the Study Outcome Measures

Scale	Mean	SD	Experiencing Problems n (%)
EPDS	16.12	6.666	1051 (74.6%)
FSS	4.30	1.50	858 (60.9%)
*PSQI*			
Component 1: Subjective sleep quality	1.39	0.887	1370 (97.2%)
Component 2: Sleep latency	1.54	1.048	
Component 3: Sleep duration	1.16	1.013	
Component 4: Sleep efficiency	2.53	0.771	
Component 5: Sleep disturbance	1.71	0.664	
Component 6: Use of sleep medication	1.35	0.916	
Component 7: Daytime dysfunction	0.68	0.813	
PSQI**	10.37	3.373	
BRS	3.18	0.73	501 (35.6%)

Data are presented as means, standard deviations (SDs), frequencies (n), and percentages (%)

*EPDS* Edinburgh Postnatal Depression Scale, *FSS* Fatigue Severity Scale, *PSQI* Pittsburgh Sleep Quality Index, *BRS* Brief Resilience Scale

**The global score of PSQI used in the analysis

### The correlations between the study variables

Table 4 illustrates the correlation matrix between the study variables. The results showed a positive correlation between EPDS scores and FSS and PSQI scores (r = 0.344 and r = 0.447 respectively; *p* value < 0.05), indicating that the higher the FSS and PSQI scores, the higher the EPDS score and vice versa. The correlation between the EPDS and BRS scores is negative (r = −0.530, *p* value < 0.05), indicating that the higher the BRS score, the lower the EPDS score and vice versa.

**Table 4:** Pearson’s Correlation Matrix of the Study Variables

	EPDS	FSS	PSQI	BRS
EPDS	1			
FSS	0.344*	1		
PSQI	0.447*	0.334*	1	
BRS	−0.530*	−0.330*	−0.364*	1

*EPDS* Edinburgh Postnatal Depression Scale, *FSS* Fatigue Severity Scale, *PSQI* Pittsburgh Sleep Quality Index, *BRS* Brief Resilience Scale

*p value < 0.05

### The predictors of EPDS

**Fig. 1:**
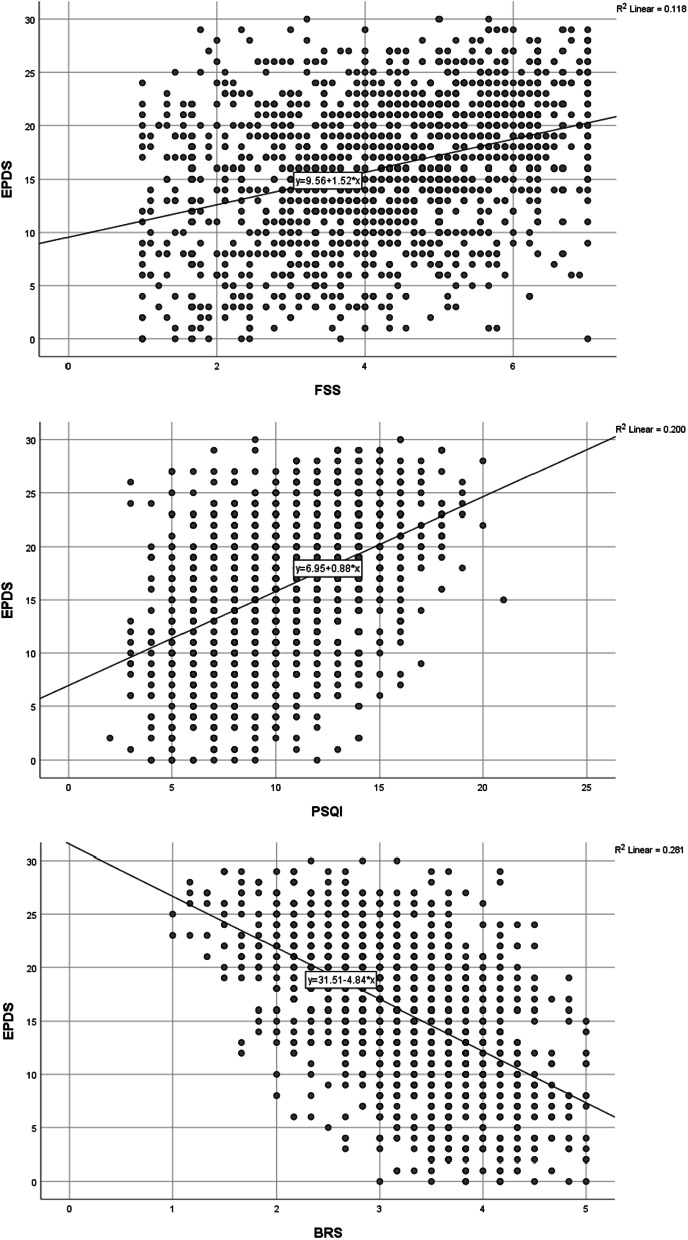
Scatter plot demonstrating the relationship between EPDS scores and FSS, PSQI, and BRS scores

Table 5 shows the simple linear regression analysis for each predictor, FSS, PSQI, and BRS scores, with regard to EPDS scores. The analysis showed a significant relationship between each predictor’s scores and EPDS scores (all *p* values < 0.05). The R^2^ of each predictor showed a strong association with EPDS scores (FSS R^2^ = 0.118; PSQI R^2^ = 200; BRS R^2^ = 281, all *p* value < 0.05) (Fig. 1). The R^2^ of each predictor in the simple linear regression analysis indicated an independent explanation of the variance in EPDS scores, with the FSS explaining 11%, the PSQI explaining 20%, and the BRS explaining 28%.

**Table 5:** Simple Linear Regression Analysis between EPDS scores and BRS/FSS/PSQI scores (N = 1409)

Dependent variable	Predictors	Coefficient	B	SE	β	t	P-value	95% CI		**R^2^
								Lower	Upper	
EPDS	FSS	^a^constant	9.561	0.506	–	18.894	0.000*	8.48	10.64	0.118
		^b^(slope)	1.525	0.111	0.344	13.731	0.000*	1.3	1.75	
	PSQI	^a^constant	6.954	0.514	–	13.534	0.000*	5.91	8	0.200
		^b^(slope)	0.884	0.047	0.447	18.758	0.000*	0.79	0.98	
	BRS	^a^constant	31.508	0.674	–	46.755	0.000*	30.33	32.69	0.281
		^b^(slope)	−4.836	0.206	−0.53	−23.427	0.000*	−5.22	−4.46	

*EPDS* Edinburgh Postnatal Depression Scale, *FSS* Fatigue Severity Scale, *PSQI* Pittsburgh Sleep Quality Index, *BRS* Brief Resilience Scale

*p ≤ 0.05 is significant; B: unstandardized beta “regression coefficient”; β: standardized beta

**R−Squared

Table 6 shows the multiple linear regression analysis of the FSS, the PSQI, the BRS, and the EPDS scores. The analysis shows significant relationships between EPDS scores and FSS, PSQI, and BRS scores (all *p* values < 0.05). The data showed a strong overall relationship in the regression model between the predictors and EPDS scores (R = 0.61, *p* value < 0.05). The R^2^ of 0.37 indicates that the predictors explain 37% of the variance in EPDS scores. We added the mother’s BMI and child’s age, smoking status, having a chronic disease, and taking anti-depressant, which were statistically correlated with EPDS scores (*p* value < 0.05), to the multiple linear regression model to control for their influence. We also included the mother’s age, and the full term of pregnancy as they could play significant roles in the scores of the study’s dependent variable. The association remained significant in the regression model (Table 7) after adjusting for mother’s age, mother’s BMI, child’s age, smoking status, full-term pregnancy, having a chronic disease, and taking anti-depressant (R = 0.621, *p* value < 0.05). The R^2^ of 0.39 indicates that the predictors explain 39% of the variance in EPDS scores.

**Table 6:** Multiple Linear Regression Analysis of the FSS, the PSQI*,* the BRS, and the EPDS scores (N = 1409)

Dependent variables	Predictors	B	SE	β	t	P-value	VIF	Overall Model P-value	95% CI		**R^2^
									Lower	Upper	
EPDS	(Constant)	19.737	1.066	–	18.521	0.000*	–	0.000	17.65	21.83	0.37
	FSS score	0.561	0.103	0.127	5.467	0.000*	1.193		0.36	0.76	
	PSQI score	0.518	0.046	0.262	11.167	0.000*	1.225		0.43	0.61	
	BRS score	−3.584	0.214	−0.393	−16.754	0.000*	1.222		−4.00	−3.16	

*VIF* Variance Inflation Factor, *EPDS* Edinburgh Postnatal Depression Scale, *FSS* Fatigue Severity Scale, *PSQI* Pittsburgh Sleep Quality Index, *BRS* Brief Resilience Scale

*p ≤ 0.05 is significant; B: unstandardized beta “regression coefficient”; β: standardized beta

**R−Squared

**Table 7:** Multiple Linear Regression Analysis of the FSS, the PSQI*,* the BRS, and the EPDS scores with covariates (N = 1409)

Dependent variables	Predictors	B	SE	β	t	P-value	VIF	Overall Model P-value	95% CI		**R^2^
									Lower	Upper	
EPDS	(Constant)	18.404	1.469	−	12.524	0.000*	−	0.000*	15.521	21.287	0.39
	FSS	0.547	0.102	0.123	5.353	0.000*	1.208		0.347	0.748	
	PSQI	0.528	0.047	0.267	11.343	0.000*	1.261		0.437	0.619	
	BRS	−3.420	0.215	−0.375	−15.881	0.000*	1.265		−3.842	−2.997	
	Mother’s age	−0.028	0.029	−0.021	−0.951	0.342	1.091		−0.086	0.030	
	Mother’s BMI	0.029	0.029	0.022	1.013	0.311	1.078		−0.027	0.085	
	Child age (months)	0.177	0.034	0.111	5.178	0.000*	1.041		0.110	0.245	
	Smoking status	0.844	0.656	0.027	1.287	0.198	1.011		−0.442	2.130	
	Full term pregnancy	−0.797	0.471	−0.036	−1.693	0.091	1.008		−1.720	0.127	
	Having chronic diseases	0.058	0.468	0.003	0.124	0.901	1.024		−0.860	0.976	
	Taking anti-depressants	1.685	0.903	0.040	1.866	0.062	1.040		−0.086	3.457	

*VIF* Variance Inflation Factor, *EPDS* Edinburgh Postnatal Depression Scale, *FSS* Fatigue Severity Scale, *PSQI* Pittsburgh Sleep Quality Index, *BRS* Brief Resilience Scale, *BMI* Body Mass Index

*p ≤ 0.05 is significant; B: unstandardized beta “regression coefficient”; β: standardized beta

**R−Squared

The estimated unstandardized B-coefficients of the FSS ≈ 0.6, indicating that, as the FSS score increases by 1 point, the EPDS score increases by 0.6 points. The unstandardized B-coefficient of the PSQI was 0.5, which means that, as PSQI scores increase by 1 point, the EPDS score increases by 0.5 points. The B-coefficient of the BRS was −3.4, meaning that when BRS scores increase by 1 point, EPDS scores decrease by 3.4 points. The data further revealed that standardized β-coefficients indicate that the importance of the predictors in predicting the EPDS scores is as follows: the BRS (β = −0.375), the PSQI (β = 0.267), and the FSS (β = 0.123).

## Discussion

The overarching aim of the current study was to determine the relationship between fatigue, sleep quality, resilience, and the risk of PPD development. Further investigation was conducted to explore the importance of resilience in predicting PPD symptoms in tandem with fatigue and sleep quality. During the postpartum period, most of our participants reported a high risk of PPD, fatigue, and sleep issues. Approximately one third of our participants reported a low level of resilience. The data revealed that the risk of PPD was positively related with the level of fatigue and sleep quality and negatively related with the level of resilience. The study results indicate that those factors are potential predictors of the risk of PPD development. Moreover, those relationships remain significant even after adjusting for the confounding effect of demographic characteristics that could also be significant predictors of a high risk of PPD. Interestingly, resilience was found to be the strongest predictor of PPD symptoms in our sample.

The EPDS, using a cutoff of 12 or more, indicated that 75% (n = 1051) of the study participants reported a high risk of PPD. These results are higher than those reported previously in the literature related to the prevalence of postpartum depressive symptoms. According to a systematic review published in 2005, the prevalence range for major or minor depression among mothers is from 6.5 to 12.9% in the months following delivery. However, when research on the prevalence of such during various postpartum periods was all combined, it was found that 19.2% of women had a major depressive episode in the first three months after giving birth [60]. Additionally, a cross-sectional study conducted in 2008 on a group of new mothers receiving routine follow-up at an urban maternity clinic in Turkey determined that, out of the 187 women included in the study, the EPDS revealed that 54 mothers (28.1%) had a score of more than 12, while 133 (71.1%) had a score of 12 [61]. Also, our results showed remarkable differences, in terms of the prevalence of PPD symptoms, as compared to those reported recently among postpartum women in Sri Lanka. Of the 975 participants in the Sri Lankan study, the prevalence of mothers with an EPDS score of 9 or more was found to be 9.4% (n = 92), and 5.6% (n = 55) had an EPDS score of 10 or more. An EPDS score of 12 or more was seen in 2.1% (n = 20) of the participants [62]. Another study, involving 124 participants in the US, also found that only 20% of participants scored 10 or higher on the EPDS, indicating a high risk for depression [63]. This discrepancy in percentages could be attributed to the life modes/cultures of the various countries, including mothering responsibilities and coping styles. It could also be attributed to differences in the cut-off points used. Furthermore, several socio-demographic, socio-economic, and health-/childbirth-related factors could contribute to these differences in PPD symptom prevalence between populations in different studies.

According to the FSS, our results showed that 61% (n = 858) of our participants reported PPF. Our results are similar to those of a study conducted on fatigue and PPD in 2018, in which 87% of women reported fatigue severity above the suggested clinical cut-off (≥ 36) [64]. Also, the reported level of fatigue among our study participants is in agreement with the results published by Badr and Zauszniewski [65]; they reported that fatigue prevalence was as high as 64% among new mothers during the postpartum period. Another study, published in 2002, showed that fatigue prevalence during the antepartum period was 20% and that this climbed to 50–64% immediately after the postpartum period [66]. The high prevalence of fatigue among mothers in our study could be explained by physiological changes, cultural issues, and family commitments [66]. Also, it has been shown that a sudden drop in certain hormones may play a role in the fatigue reported by mothers [12]. In addition, having a new baby with irregular sleeping patterns [67] who cries without explanation and frequently wakes up for feeding [68] can contribute to mothers’ reported fatigue after delivery. Poor sleep among mothers during this critical transitional period due to the abovementioned factors related to the newborn—along with the responsibility of taking care of household chores, other siblings’ responsibilities, or even job-related responsibilities [69]—may collectively lead to fatigue and exhaustion. However, investigating the interactions between study variables and/or the factors that contributed to the reported level of fatigue among our participants was beyond the scope of this research. Further study regarding this matter is warranted.

In regard to the PSQI, a very high percentage of our participants (97%) reported not sleeping well. This trend of sleeplessness is similar to results published in 2022, in which nearly 60% of postpartum women reported poor sleep quality two months after delivery [70]. Additionally, the findings of a study by Ko and Lee [71] that aimed to improve sleep quality among postpartum women revealed that more than 95.4% of women reported sleep disturbances. Such a high prevalence of poor sleep could be attributed to several factors. A study that examined infants’ frequent nocturnal awakenings and night-time feedings found that poor sleep quality was a common problem throughout the postpartum period [72]. Interestingly, unlike with sleep quality, it has been shown that breastfeeding could be a protective factor against PPD. Borra et al. [73], revealed that the risk of PPD was lower in breastfeeding mothers as compared with those who were not intending to breastfeed. This relationship between breastfeeding and depressive symptoms has been attributed to the regulation of diurnal basal cortisol secretion (hypothalamic pituitary adrenal (HPA) axis) [74–78]. However, the influence of breastfeeding during postpartum warrants further attention.

Moreover, factors such as employment, the sex of the baby, home birth, antenatal mental illness, medical issues after delivery, and PPD were positively associated with poor sleep quality [79]. Although the effect of the fear of sudden infant death syndrome (SIDS) on maternal sleep quality has not received much attention in the literature, there could be an indirect interaction in this regard. Fear of SIDS may lead to anxiety and depression, which may, in turn, impact a mother’s quality of sleep [80]. Lack of sleep can eventually lead to becoming physiologically [81], psychologically [82], and physically [83] compromised. Therefore, it is important to improve awareness and implement preventive strategies to educate mothers about the importance of sleep and the health consequences of sleep deprivation [20]. Several methods with which to promote healthy sleep have been reported in the literature. For instance, midwives and obstetricians have found that Pilates activities enhanced physical and mental health in the primigravida population during their postpartum period [84]. Also, a meta-analysis revealed that cognitive behavioral therapy for insomnia could improve both daytime mood and nocturnal sleep issues [85]. Moreover, engaging in regular, moderate-intensity gymnastic exercise to relieve stress and exhaustion and enhance sleep has been recommended [86]. In this study, we did not investigate the factors predisposing one to poor sleep or how to improve sleep. In light of the differences, in terms of cultural, health- and birth-related, or psychosocial factors between our study population and the those studied previously, further study is essential in explaining the high percentage of poor sleep reported by our sample. Also, further studies to improve sleep quality among mothers are still warranted.

Another important finding of this study was that 36% of the participants fell within the category of low resilience. To the best of the author’s knowledge, there is a paucity of research investigating resilience among mothers during the postpartum period. In one of the few studies conducted to understand the potential protective factors for perinatal women during the COVID-19 pandemic, the results showed a low level of resilience among pregnant and postpartum women [87]. However, the said study is different from our current study in that we focused on delineating the level of resilience among postpartum women and how it could contribute to the development of PPD. Additionally, a 2022 study that investigated women’s coping styles during pregnancy and their relationship to PPD revealed that women with higher educational backgrounds, higher self-esteem, or better mental resilience had higher scores for positive coping. In addition, 26.34% of postpartum women experience depression; this implies high levels of negative coping styles, which could be associated with low levels of mental resilience, as well as poor educational backgrounds and low self-esteem [88]. However, as the population studied was composed of Chinese women, they may have different lifestyles, cultures, awareness, and mentality as compared to the population used in our study. Regardless, resilience, as an emerging psychological construct among postpartum women, necessitates further investigation.

The results of this study support the hypothesis that a higher level of PPF is predictive of a high risk of PPD, which, in turn, suggests the importance of fatigue screening to identify mothers who are at risk of developing PPD. Our results are supported by those of a 2018 study in which the FSS and the Depression Anxiety Stress Scale (DASS21-D) indicated a correlation between fatigue and PPD [64]. Moreover, in a study by Bozoky and Corwin [13], a relationship between fatigue and PPD was also found. It has been stated that fatigue is the early symptom of PPD [89]. Fatigue is one of the top four factors that women believe contribute to PPD [90], as the vast majority of postpartum depressed women have been shown to feel fatigued [91, 92]. It has been widely mentioned that PPD may hinder a new mother in terms of developing her maternal role. Therefore, fatigue, as a potential predictor, should be assessed early, and rehabilitative management strategies could be implemented to reduce fatigue and thus the risk of PPD development among mothers.

The relationship between sleep quality and the risk of PPD has been examined in several studies. One study that evaluated the relationship between sleep quality and symptoms of depression found that poor sleep quality was strongly linked with depression [19]. Another study, employing the PSQI, determined that a lack of sleep increases a woman’s vulnerability to depression [70]. In addition, a study investigating sleeping time during pregnancy has shown that less sleep was associated with a higher risk of depression [93]. Moreover, a study conducted in Pennsylvania among 44 women who were 6 to 26 weeks postpartum showed a linear correlation between poor sleep quality and the severity of depression symptoms and revealed that women with depression had poorer sleep quality than women without depression [94]. The relationship between sleep and depression is demonstrated in the literature, and all the above-mentioned studies are in line with the results of our study among mothers living in Saudi Arabia. However, it has been shown that depression and sleep have reciprocal interactions, in which depression can lead to poor sleep quality and, in turn, poor sleep quality can eventually lead to depression [95]. Further studies are needed to understand the factors that strengthen or weaken this relationship.

This study also found an association between the level of resilience and the risk of PPD. We demonstrated that resilience decreased the likelihood of PPD symptoms. A 2021 study by Kinser et al. [96], which used the Connor-Davidson Resilience Scale 2 (CD-RISC 2), found that higher resilience scores on the CD-RISC 2 were related to lower ratings for symptoms of depression; however, the study was conducted during the COVID-19 pandemic, which could have generated other explanations regarding the relationship between resilience and depressive symptoms. Moreover, another study on postpartum women that used the Connor-Davidson Resilience Scale (CD-RISC) to examine the relationship between resilience and depression revealed a negative relationship [97]. There is a paucity of research examining this relationship directly among mothers in their postpartum period. Resilience, as a coping strategy, could promote mothers’ ability to handle the changes they face in their lives during this critical period. An expansion of research on this relationship and in relation to other psychological/ psychosocial factors is recommended. Researchers should also focus on adapting behaviors and acceptance strategies among mothers so as to enhance their overall health.

Several other factors could also be potential predictors of a high risk of PPD. Our analysis revealed that fatigue, sleep quality, and resilience, as evaluated by BRS, FSS, and PSQI scores, are strong predictors of EPDS scores. We also revealed that other variables could influence EPDS scores. We found that the age of the newborn contributes to the risk of PPD and that an increase of a month increases the EPDS scores. The relationship between baby age and the mother’s risk of PPD was revealed by Dol et al. [98] in a Canadian study, namely women with older infants experienced more PPD symptoms than those with younger infants. In addition, the relationship between baby age and the mother’s risk of PPD could be explained by the mediating effect of poor sleep [99]. However, the mediating and moderating variables in the relationship between predisposing factors and risk of PPD development require further studies, as there could be direct or indirect interactions between the variables.

Moreover, obesity and overweight among mothers have been shown to contribute to the risk of PPD [100]. The relationship between PPD symptoms and BMI was also explained by LaCoursiere et al., in which the results show that an increase in BMI was associated with postpartum depression symptomology in 30.8% of obese women [101]. In addition, advanced maternal age has been also shown to be linked to PPD. Those who are 40–44 years old showed higher levels of PPD than those aged 30–35 years old [102]. In addition, smoking has been shown to link with a higher risk of PPD. In a recent Japanese study, the authors revealed that women who continued smoking during pregnancy or ceased smoking after becoming pregnant had greater probabilities of developing PPD compared to those who had never smoked. They also revealed that women who quitted smoking of ≤ 5 years before getting pregnant are still at high risk of developing PPD [103].

In regard to adverse experiences of pregnancy or delivery, studies have shown the association between the adverse experiences of delivery or pregnancy and risk of PPD. It was revealed that those who experienced premature delivery are at risk of developing PPD [104, 105]. In addition, using medications such as antidepressant drugs could worsen the case and increase the risk of having PPD. Mothers might be concerned about drugs addiction, side effects, and the stigma associated with taking such drugs, which eventually may precipitate a premature discontinuation of taking the medications and therefore making the matters worse [106–111]. Chronic diseases on other hand could exacerbate the susceptibility of having depression. A large population-based study stated that those with chronic conditions are more likely to suffer from depressive symptoms when compared to those without chronic illnesses. While chronic conditions and depression co-occur, health is further impaired to a greater degree than if each occurs alone [112–114].

In this study, factors such as the mother’s age and BMI, the child’s age, smoking status, full-term pregnancy, having a chronic disease, and taking anti-depressant were all controlled for, but the relationships between fatigue, sleep, and resilience and the risk of PPD were still significant. However, investigating the other factors contributing to the risk of PPD development among our participants was beyond the scope of this study. Future studies should explore the sociodemographic and health- and childbirth-related factors affecting the risk of depression development in postpartum women.

### Study strengths and limitations

The current study’s main strength is that it is a multi-region population-based study with a large sample size that includes women who live in Saudi Arabia. However, there are also several limitations that must be acknowledged. The study included a non-probability convenience sampling method, and as with all trials based of convenience sampling, the generalizability of the results to the general population is limited. All our participants were recruited using electronic platforms, which may have resulted in selection bias, and therefore, limited the generalizability of our findings to all mothers. Future studies should consider randomized data collection or in-person interview/qualitative methods to avoid such limitation. Our results were limited because the risk of PPD, the level of PPF, sleep quality, and resilience level were self-reported, which may have led to reporting bias. Using objective measures would be more appropriate in future studies.

This study investigated the role of resilience, as one psychological predictor, on the risk of development of PPD. However, we did not collect information related to other psychological factors, such as self-efficacy, that may also have contributed to the development of PPD during the postpartum period. It was beyond the scope of the study to directly investigate the relationship between the sociodemographic/health and childbirth factors such as breastfeeding status, parity, delivery mode, regular exercise, and the risk of PPD development. Further studies to provide an overview of the potential impact of psychological, environmental, lifestyle, and health-related factors to postpartum depressive symptoms are recommended.

We also did investigate the factors contributing to the reported levels of fatigue, resilience, and sleep quality. The study design was cross-sectional; we do not have baseline data about the above-mentioned factors, and we did not investigate the levels of such, either during or even before pregnancy. Other factors, such as pain levels, were not included in the distributed questionnaire. Pain may have an influence on mothers’ risk of developing PPD during this critical period. Such relationship needs future research.

It is also worth noting that this study was limited by the lack of evidence on the relationship between the risk of PPD and resilience. Some studies have implied a positive effect on the part of mental resilience via coping styles on the risk of PPD, without addressing this topic directly, but to the authors’ knowledge, there are no available research that can support the findings related to the risk of PPD and resilience in our study. In addition, the Arabic version of the PSQI used in this study was associated with an internal consistency value (α = 0.65) that is below the threshold for acceptable reliability, which is 0.70. To ensure the internal consistency of the scale, further reliability study among Arabic population including a larger sample size is required. Finally, research concerning participation in physical activity and exercise and its effects on fatigue, sleep quality, and resilience among expectant and new mothers should also be given focused attention, as exercise has been shown to be a cost-effective intervention with which to improve psychological well-being and prevent postpartum depressive symptoms [86, 115, 116].

## Conclusions

Mothers with higher levels of fatigue, poor sleep quality, and low resilience were at high risk of developing PPD. Even though those factors were strong predictors of PPD symptoms, other factors related to mothers’ characteristics could contribute to the development of PPD and should not be overlooked. Our data suggested that resilience could be a promising coping strategy with which to buffer the development of PPD. Healthcare providers should identify these factors and thus set better rehabilitation goals to improve overall maternal health.

## Data Availability

The data is not publicly available because further research is being conducted and more manuscripts are being prepared. Data for the present study will be made accessible upon reasonable request from the principal investigator or corresponding author.

## Abbreviations

BMI: Body Mass Index
PPD: Postpartum depression
PPF: Postpartum fatigue
EPDS: Edinburgh Postnatal Depression Scale
FSS: Fatigue Severity Scale
PSQI: Pittsburgh Sleep Quality Index
BRS: Brief Resilience Scale
WHO: World Health Organization
CD-RISC: Connor-Davidson Resilience Scale
DASS21-D: Depression Anxiety Stress Scale
